# Erythrocytosis caused by giant chromophobe renal cell carcinoma: a case report indicating a 9-year misdiagnosis of polycythemia vera

**DOI:** 10.1186/s40880-017-0238-z

**Published:** 2017-09-06

**Authors:** Renbo Guo, Yiran Liang, Lei Yan, Zhonghua Xu, Juchao Ren

**Affiliations:** 1 Department of Urology, Shandong Cancer Hospital Affiliated to Shandong University, Jinan, 250117 Shandong P. R. China; 2 Department of Breast Surgery, QiLu Hospital, Shandong University, Jinan, 250012 Shandong P. R. China; 3 Department of Urology, QiLu Hospital, Shandong University, Jinan, 250012 Shandong P. R. China

**Keywords:** Chromophobe renal cell carcinoma, Polycythemia vera, Erythrocytosis, Misdiagnosis

## Abstract

**Background:**

Erythrocytosis, a rare paraneoplastic syndrome, generally occurs in patients with clear cell renal cell carcinoma and has never been reported in patients with chromophobe renal cell carcinoma.

**Case presentation:**

We report a case of a young man suffering from a giant (22-cm) mass on his left kidney. Because of a history of polycythemia vera, the patient had been treated for the condition for 9 years. Radical nephrectomy was successfully performed, and the postoperative pathologic examination confirmed a diagnosis of chromophobe renal cell carcinoma. Unexpectedly, the symptom of erythrocytosis disappeared after the surgery. Further examination and analysis were performed, and we finally attributed his erythrocytosis to chromophobe renal cell carcinoma.

**Conclusions:**

Chromophobe renal cell carcinoma could cause erythrocytosis, but the clear-cut mechanism needs further research. Secondary erythrocytosis such as those related with renal tumors should be taken into consideration during the diagnosis of polycythemia vera.

## Background

Renal cell carcinoma (RCC) is the most common renal malignancy in adults. Chromophobe renal cell carcinoma (chRCC) is a rare subtype of RCC that was first described in 1985 [1]. Giant (>20 cm) chRCC in young patients (<30 years old) has rarely been reported [1, 2].

Erythrocytosis can be classified as either primary or secondary [3]. Secondary erythrocytosis is mainly caused by conditions resulting in increased erythropoietin (EPO) production, including tumor or tissue production [3]. Polycythemia vera (PV) as a primary condition is a type of clonal disorder of bone marrow stem cells which is often caused by a mutation in exon 12 of the janus kinase 2 (*JAK2*) tyrosine kinase gene [4, 5]. The mutation of the gene increases the phosphorylation activity of JAK2, promotes spontaneous cell growth, and induces erythrocytosis [6].

Erythrocytosis related with RCC is a secondary condition and is a rare event, occurring in no more than 5% RCC patients [7]; almost all cases of erythrocytosis occur in patients with clear cell renal cell carcinoma (ccRCC) [8]. Flank discomfort or pain, gross hematuria, flank mass, and weight loss can be observed in patients with chRCC; other symptoms include renal dysfunction, proteinuria, and pain from metastatic sites [9–11]. chRCC with symptoms of erythrocytosis is extremely uncommon.

Here, we present a case of a young man suffering from giant chRCC with erythrocytosis who had been unfortunately misdiagnosed with PV for 9 years.

## Case presentation

A 29-year-old man was referred to the Department of Urology in QiLu Hospital on May 5, 2015 for diagnosis and treatment of a giant abdominal mass, which was discovered incidentally 1 month earlier. The patient had a medical history of PV associated with symptoms of splenomegaly and flush for 9 years. The patient had been admitted to a local hospital because of flush in 2006. The physical examination indicated splenomegaly, peripheral blood test and bone marrow trephine biopsy indicated erythrocytosis, and the result of p210 breakpoint cluster region-Abelson (BCR-ABL) fusion gene examination was negative. The patient was then diagnosed with PV. The results of peripheral blood tests of the patient remained abnormal; as a result, the patient was treated with intermittent phlebotomy therapy at the local hospital for 9 years. He also complained of progressive swelling of the abdomen, but the sign had always been ignored because it was considered to be related to splenomegaly. The patient had no symptoms, such as hematuresis, headache, fever, cough, and pain, suggesting any other syndromes.

**Fig. 1:**
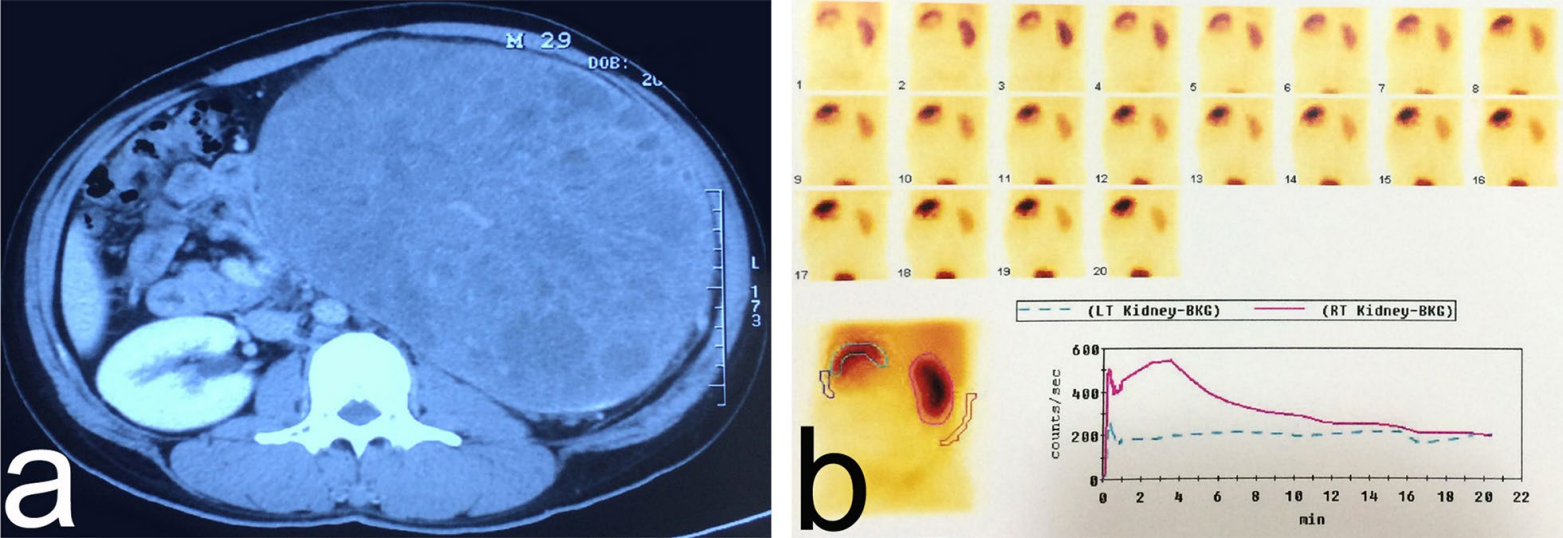
Computed tomography and emission computed tomography of a 29-year-old man with a giant abdominal mass. **a** Computed tomography of the abdomen on May 6, 2015 before the surgery demonstrates a giant (22 cm × 16 cm) solid mass in the left kidney without necrosis or calcifications. Heterogeneous enhancement can be seen during the arterial phase, and normal renal tissues are seriously squeezed. **b** Emission computed tomography reveals mild-to-moderate damage of the left kidney and normal function of the right kidney

At QiLu Hospital, the physical examination revealed a solid painless giant mass on the left side of the abdomen. Computed tomography of the abdomen and pelvis on May 6, 2015 showed a giant mass with a maximum diameter of 22 cm located in the middle-to-upper pole of the left kidney. The normal renal tissue of the left kidney was seriously constricted, and the ipsilateral renal pelvis of the right kidney showed hydronephrotic changes (Fig. 1a). Renal emission computed tomography (ECT) revealed mild-to-moderate damage in the left kidney (Fig. 1b). The results of peripheral blood test were consistent with the diagnosis of PV: a red blood cell count of 6.44 × 10^12^/L, a hemoglobin level of 22.2 g/dL, and a hematocrit level of 62.8%.

Following the routine examination, percutaneous renal biopsy under ultrasound guidance was performed on May 13, 2015 at the strong request of the patient to define the diagnosis. The histologic result indicated a diagnosis of RCC, but the specific subtype could not be clearly confirmed.

Treatment was initiated on May 15, 2015 with phlebotomy therapy to reduce the level of erythrocytosis and the risk of surgery. Following the hematologist’s advice, 8 sessions of bloodletting (400 mL each time, once a week) were performed over the following 2 months, and aspirin at a dose of 100 mg/day was administered at the same time to prevent thrombogenesis. The patient’s hemoglobin and hematocrit levels were controlled within normal limits before surgery.

**Fig. 2:**
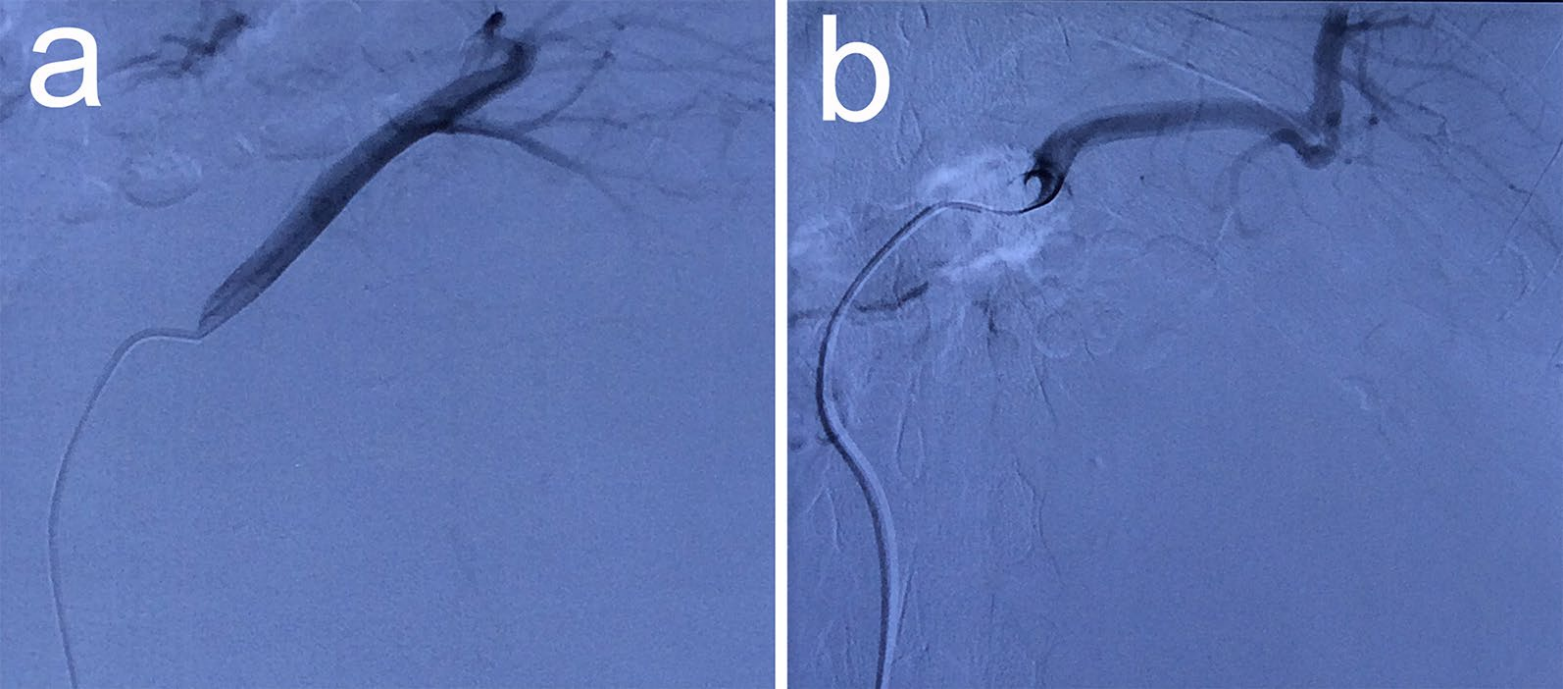
Preoperative angiography and embolization of the left renal artery. Embolization of the left renal artery was performed on June 30, 2015 to diminish the volume of the mass and to reduce the blood loss during surgery. **a** The left renal artery before the embolization. **b** The embolized left renal artery

**Fig. 3:**
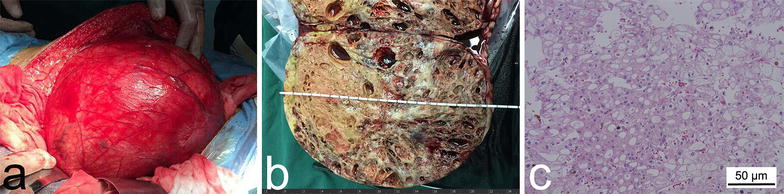
Gross appearance and histopathologic examination of the tumor. **a** Appearance of the giant tumor on the left kidney during the surgery. A great amount of circuitous blood vessels are extensively distributed on the surface of the mass. **b** Postoperative gross pathologic examination of the specimen. The specimen measures 20.5 cm × 16 cm × 15 cm. Multiple tiny cysts of various sizes appear on the light brown or tan maximum cross-section of the giant solid tumor. **c** Pathologic examination of the lesion with hematoxylin–eosin staining (×200) reveals large cells with finely reticulated cytoplasm and small cells with abundant eosinophilic cytoplasm and perinuclear clearing

To prevent serious intraoperative bleeding, a selective embolization of the left renal artery was also performed on June 30, 2015 (Fig. 2). Afterwards, an open radical left nephrectomy was performed. No obvious infiltration or local metastasis around the tumor was found during the surgery (Fig. 3a). The total operation time (including the embolization) was 175 min. Intraoperative blood loss was approximately 500 mL, and no blood transfusion was performed.

**Fig. 4:**
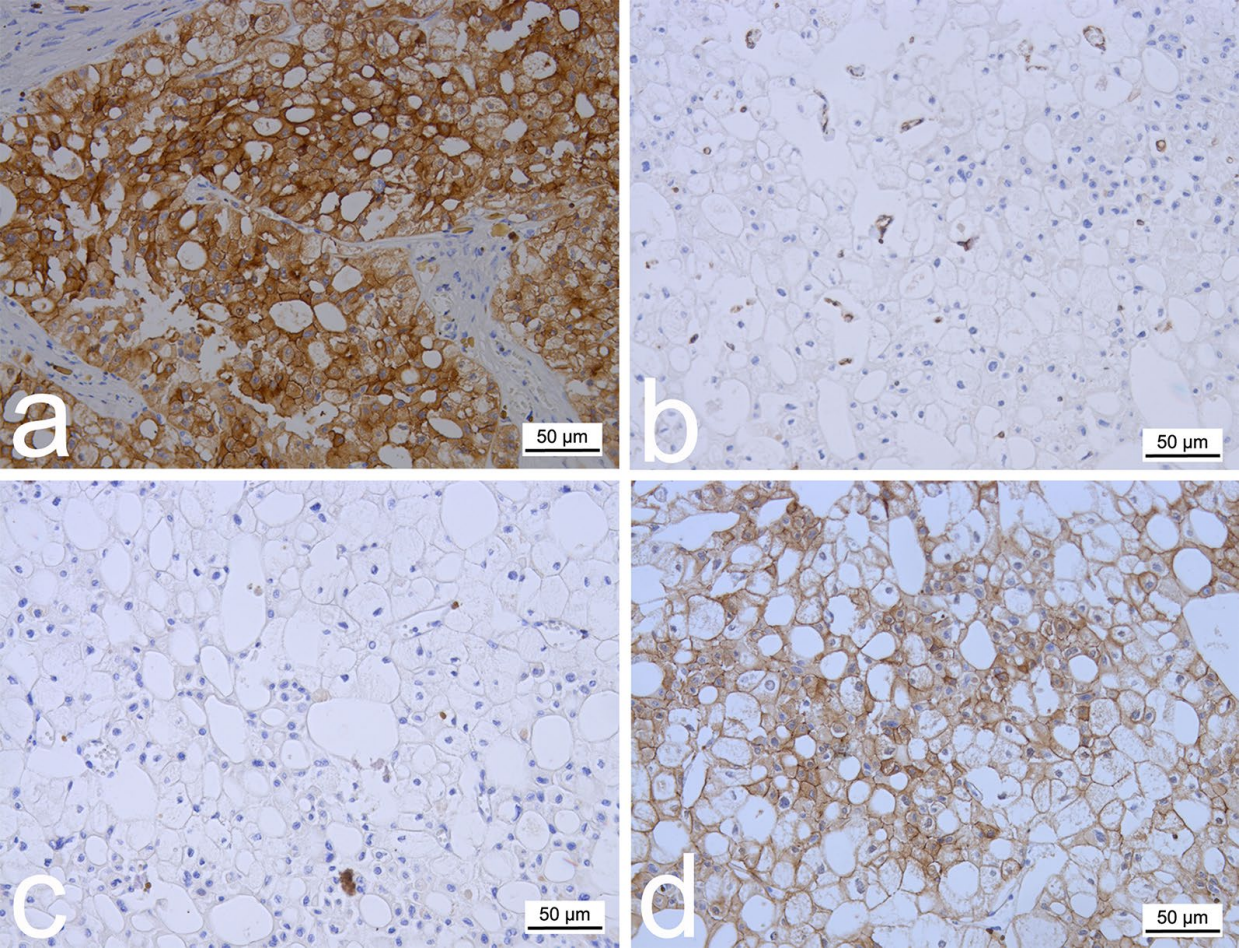
Immunohistochemical examination of the tumor with specific antibodies. Immunohistochemical staining of the tumor section shows **a** strong positivity for cytokeratin 7 in both the cytoplasm and membrane, **b** negative Vimentin expression, **c** negative CD10 expression in both the cytoplasm and membrane, and **d** middle to strong positivity for erythropoietin (EPO) in the cytoplasm. The results confirmed the diagnosis of chromophobe renal cell carcinoma and EPO production of the tumor

The pathologic examination of the gross specimen revealed an encapsulated solid mass measuring 20.5 cm × 16 cm × 15 cm, with multiple tiny cysts of various sizes in the central part of the tumor (Fig. 3b). Large cells with finely reticulated cytoplasm and small cells with abundant eosinophilic cytoplasm and perinuclear clearing were found using hematoxylin–eosin staining (Fig. 3c). Immunohistochemical staining was also performed with paraffin-embedded samples, which were positive for cytokeratin 7 (CK7) and negative for Vimentin and CD10 (Fig. 4a–c). Considering the combination of the above results, the patient was finally diagnosed with stage pT2bN0M0 chRCC (according to the 2010 American Joint Committee on Cancer TNM staging system). A test for the *JAK2* V617F mutation was performed, which returned a negative result, excluding the diagnosis of PV. Immunohistochemical staining with EPO-specific antibody was also performed, which was positive (Fig. 4d). Therefore, we confirmed that the erythrocytosis was caused by the tumor and was a type of paraneoplastic syndrome of chRCC.

The upper and lower abdominal drains were removed separately 5 and 10 days later, and the patient was discharged on the 15th postoperative day. His 30-day postoperative hemoglobin level was 13.4 g/dL, and his hematocrit level was 40.0%.

**Fig. 5:**
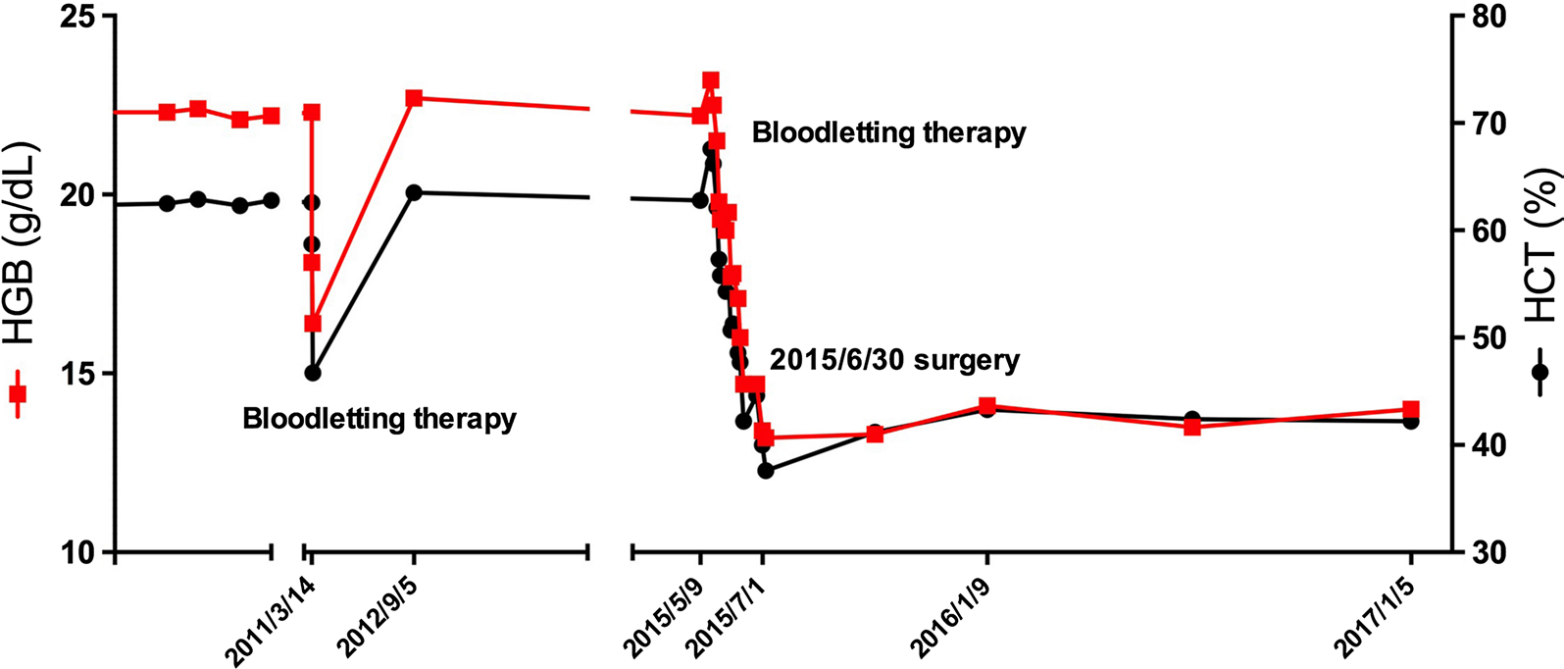
Changes in the patient’s serum hemoglobin (HGB) and hematocrit (HCT) levels before and after surgery. The patient’s HGB and HCT remained at high levels before the surgery, and he was treated with intermittent bloodletting therapies at the local hospital for 9 years. One of the bloodletting therapies was taken on March 14, 2011, and his HGB and HCT declined after the bloodletting therapy. During the time of the two breaks on the horizontal axis, the bloodletting therapy and blood examination was continued, however it was not recorded. His HGB and HCT were controlled within normal levels with bloodletting therapy before surgery at QiLu Hospital. After being discharged from QiLu hospital, the patient’s HGB and HCT levels remained normal without any adjuvant therapy during the 18 months of follow-up.

During the 18 months of follow-up until January 5, 2017, no evidence of disease recurrence or metastasis was identified. The patient’s blood test index was still within normal limits without any adjuvant therapies (Fig. 5).

## Discussion

In the present case, immunohistochemical staining was performed for CK7, Vimentin, CD10, and EPO. The results were consistent with a diagnosis of chRCC according to the relevant reports and distinguished the condition from both ccRCC and renal oncocytoma [12–15]. In addition, plasma from the tumor cells was also EPO-positive. In combination with the patient’s recovery from “PV”, we confirmed that his erythrocytosis was caused by chRCC due to EPO production.

According to the patient’s medical history of erythrocytosis, we speculated that his renal tumor had a certain volume 9 years ago but was likely concealed by the patient’s symptoms of splenomegaly and failed to draw the attention of the physician. As a result, the patient was misdiagnosed with PV without any imaging examinations. During the subsequent 9 years, even though his abdomen was progressively swelling, the patient did not have any imaging examinations because the swelling was considered to be due to splenomegaly.

Renal artery embolization (RAE) was performed before radical nephrectomy in this case. During the surgery, we observed an abundant and complex blood supply traveling through the distended vessels on the surface of the tumor; however, the intraoperative blood loss was controlled within 500 mL without blood transfusion. There were also no complications caused by preoperative RAE, such as small groin hematomas or post-infarction syndromes including nausea or flank pain, as mentioned in a previous report [16].

It has been reported that RCC is the most common cancer causing erythrocytosis [8], especially the ccRCC subtype [8, 17]; other subtypes are rarely reported. Paraneoplastic syndromes of erythrocytosis have never been reported in chRCC. In addition, the facts that the chRCC occurred at such a giant volume and at such a young age without any metastasis, necrosis, or recurrence and was misdiagnosed as PV for 9 years make this case fairly unique.

As has been reported, secondary erythrocytosis occurs when factors outside of the bone marrow, such as tumors or other abnormal organs, stimulate EPO production [18]. To distinguish primary and secondary conditions, “no cause of secondary erythrocytosis” was added to the diagnosis criteria for the clinical practice guidelines of PV [19]. In recent years, it has generally been accepted that the *JAK2* mutation presents in most PV patients [20]. As a result, the test for the *JAK2* mutation was added to the 2008 World Health Organization criteria for the diagnosis of PV [21–23]. However, *JAK2*-negative PV cases have also been reported [22–26]. Therefore, the diagnosis of PV still requires comprehensive examination and consideration in clinical practice.

In consideration of the present case and the factors mentioned above, some measures can be adopted during the diagnosis of erythrocytosis or erythrocytosis combined with cancer. First, imaging examinations, such as ultrasound or computed tomography, should be performed, especially for patients with signs such as abdominal distention or splenomegaly to exclude secondary erythrocytosis. Additionally, patients with erythrocytosis for whom secondary factors have already been ruled out should be tested for the *JAK2* mutation if possible to confirm the PV diagnosis.

RAE was initially developed in the 1970s [27]. It was performed to treat renal cancer and has been demonstrated to be a safe and effective technique with several decades of experience [28]. However, whether preoperative RAE is indeed beneficial for survival is still controversial [16, 28–30]. From our perspective, in the present case, preoperative RAE reduced the tumor blood supply, helped earlier ligation of the vessel, shortened the operation time, and played a positive role without any adverse effects. However, just one case is not yet illustrative of its benefit; further studies are needed to evaluate the advantages and disadvantages of preoperative RAE.

Certainly, this report still has limitations. For instance, erythrocytosis caused by EPO production was found in this case. The mechanisms of EPO production were not explored in this report and still require further research. The patient’s follow-up time was just 18 months. A longer follow-up is also needed to assess the patient’s prognosis and occurrence of relapse.

## Conclusions

In conclusion, chRCC associated with erythrocytosis is a unique event. The explicit mechanism of this condition still needs further study. Secondary erythrocytosis should be taken into consideration while diagnosing PV. Imaging examination and *JAK2* testing should be performed to avoid a misdiagnosis. Preoperative RAE played a positive role in the present case, but it is a controversial method that requires further evaluation.

## Abbreviations

RCC: renal cell carcinoma
chRCC: chromophobe renal cell carcinoma
PV: polycythemia vera
EPO: erythropoietin
CK7: cytokeratin 7
CD10/MME: membrane metallo-endopeptidase
RAE: renal artery embolization
